# An early biomarker and potential therapeutic target of *RUNX* 3 hypermethylation in breast cancer, a system review and meta-analysis

**DOI:** 10.18632/oncotarget.13125

**Published:** 2016-11-04

**Authors:** De-guo Lu, Ying-mei Ma, Ai-ju Zhu, Yun-wei Han

**Affiliations:** ^1^ Clinical Laboratory, Linyi People’s Hospital, Linyi, Shandong, P.R. China; ^2^ Clinical Laboratory, Linyi Chest Hospital, Linyi, Shandong, P.R. China; ^3^ Department of ophtalmology, Linyi People’s Hospital, Linyi, Shandong, P.R. China; ^4^ Department of Oncology, The Affiliated Hospital of Southwest Medical University, Luzhou, Sichuan, P. R. China

**Keywords:** RUNX3, methylation, odds ratio, prognosis, drug target

## Abstract

Runt-related transcription factor 3 (*RUNX3*) methylation plays an important role in the carcinogenesis of breast cancer (BC). However, the association between *RUNX3* hypermethylation and significance of BC remains under investigation. The purpose of this study is to perform a meta-analysis and literature review to evaluate the clinicopathological significance of *RUNX3* hypermethylation in BC. A comprehensive literature search was performed in Medline, Web of Science, EMBASE, Cochrane Library Database, CNKI and Google scholar. A total of 10 studies and 747 patients were included for the meta-analysis. Pooled odds ratios (ORs) with corresponding confidence intervals (CIs) were evaluated and summarized respectively. *RUNX3* hypermethylation was significantly correlated with the risk of ductal carcinoma *in situ* (DCIS) and invasive ductal carcinoma (IDC), OR was 50.37, *p* < 0.00001 and 22.66, *p* < 0.00001 respectively. Interestingly, the frequency of *RUNX3* hypermethylation increased in estrogen receptor (ER) positive BC, OR was 12.12, *p* = 0.005. High *RUNX3* mRNA expression was strongly associated with better relapse-free survival (RFS) in BC patients. In summary, *RUNX3* methylation could be a promising early biomarker for the diagnosis of BC. High *RUNX3* mRNA expression is correlated to better RFS in BC patients. RUNX3 could be a potential therapeutic target for the development of personalized therapy.

## INTRODUCTION

Breast cancer (BC) is the most frequently diagnosed cancer and the leading cancer related death for women worldwide, with 232,340 new cases every year [1]. Carcinogenesis in breast is a linear multi-step process which starts as flat epithelial atypia (FEA), progresses to atypical ductal hyperplasia (ADH), advances to ductal carcinoma in situ (DCIS) and invasive ductal carcinoma (IDC). The most common cause of death in BC is invasive malignancy. Therefore, it is critical to identify an early detection biomarker to predict the progression of BC [2].

The studies of molecular mechanism have demonstrated that the carcinogenesis involves the accumulation of various genetic alterations including loss of tumor suppressor genes and amplification of oncogenes [3]. RUNX (Runt-related transcription factor) family of genes including RUNX1, RUNX2 and RUNX3, encode transcription factors which bind DNA by partnering with the cofactor, CBFβ/PEBP2β (core-binding factor-beta subunit/polyomavirus enhancer-binding protein 2 beta subunit). The complex regulates the growth, survival and differentiation *via* a few essential transcription factors [4]. RUNX3 plays an important role in gastric epithelial growth [5], development of dorsal root ganglia [6–7] and T-cell differentiation [8], and has a principle role in the regulation of cell proliferation, cell death, angiogenesis, as well as invasion [9–10]. RUNX3 was first reported as a tumor suppressor in gastric cancer because of the causal link between the loss of RUNX3 and gastric carcinogenesis [5]. Since then, RUNX3 has been observed as a suppressor that is inactivated in a variety of pre-invasive and invasive tumor including BC [11]. RUNX3 protein regulates the growth-suppressive effects of transforming growth factor-β (TGF- β) by associating with SMAD, a downstream protein in the signaling pathway [12]. Recently, RUNX3 has been reported to attenuate Wnt signaling by directly suppressing β-catenin/TCF4 in colon cancer and gastric cancer [13]. Previous evidences in cell lines, knockout animals, and primary human cancer tissues have indicated that RUNX3 as a suppressor is inactivated in BC by reduced copy number [14], protein mislocalization [10, 15], hemizygous deletion and promoter hypermethylation [16–18]. However, the role of RUNX3 as a tumor suppressor in the progression and prognosis of BC remains unclear due to the small sample size of individual studies. We conducted a meta-analysis which increases the sample size and thus the power, to investigate the significance of RUNX3 hypermethylation in the progression and prognosis of BC.

## RESULTS

**Figure 1:**
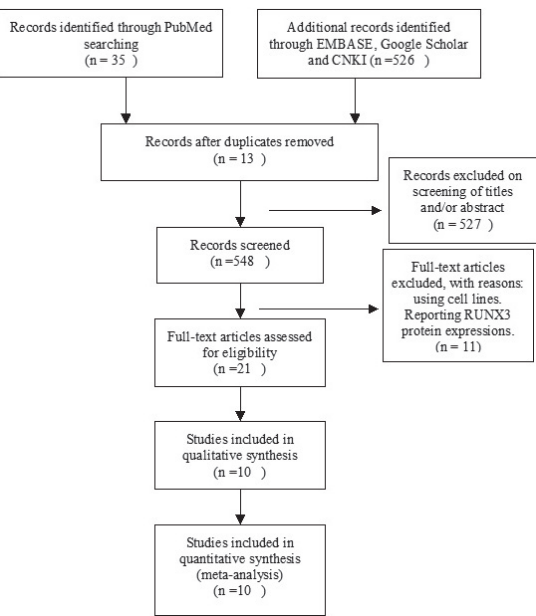
Schematic flow diagram for selection of included studies

10 studies were included after screening 862 studies by two authors (Figure 1). The following variables were listed: first author, published year, country, ER status, RUNX3 methylation status and patient progressions (Table 1).

**Table 1:** Main characteristics of included studies

Author	Year	Country	Normal		Benign		DCIS		IDC				Methylation-site
			Methylation	Ex-pression	Methylation	Expression	Methylation	Expression	Methylation	Expression	ER Status (-/+)	Methods	
Park[38]	2011	Korea	0/30		0/30		17/35		24/50		17/68	Methy light	Promoter CpG islands
Subramaniam[39]	2010	Singapore	1/30	28/30	0/20	99/101	15/20	3/20	16/20	2/20	N/A	MSP IHC	Promoter CpG islands
Subramaniam [10]	2009	Singapore	1/10	9/10			13/17	3/17	16/21	2/23	N/A	MSP IHC	Promoter CpG islands
Lau[15]	2006	Singapore	0/20	20/20					23/44	0/44	N/A	MSP IHC	Promoter CpG islands
Du[40]	2010	China			0/15				19/40		N/A	MSP	Promoter CpG islands
Suzuki[11]	2005	Japan									11/22	MSP	Promoter CpG islands
Li[41]	2013	China	1/12						25/48		N/A	MSP	Promoter CpG islands
Qiao[42]	2012	China	4/60						35/60		N/A	MSP	Promoter CpG islands
Tian[43]	2010	China	6/56						31/56		N/A	MSP	Promoter CpG islands
Jiang[16]	2008	China	0/15	15/15					13/15	0/15	48/40	MSP IHC	Promoter CpG islands

MSP: Methylation-Specific Polymerase Chain Reaction

**Figure 2:**
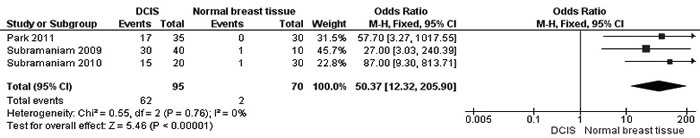
Forest plot for *RUNX3* methylation in DCIS and normal breast tissue The squares represent the weight of individual study in the meta-analysis, the line width indicates the corresponding 95% CI, The diamond represents the pooled OR, and the width of diamond indicates 95% CI. Abbreviations: M-H: Mantel-Haenszel, CI: Confidence Interval, DCIS: Ductal Carcinoma *In Situ*.

**Figure 3:**
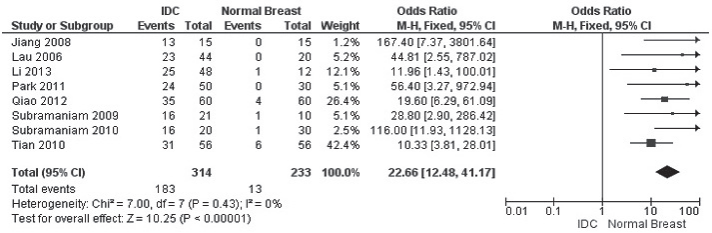
Forest plot for *RUNX3* methylation in IDC and normal breast tissue The squares represent the weight of individual study in the meta-analysis, the line width indicates the corresponding 95% CI, The diamond represents the pooled OR, and the width of diamond indicates 95% CI. Abbreviations: M-H: Mantel-Haenszel, CI: Confidence Interval, IDC: Invasive Ductal Carcinoma.

**Figure 4:**
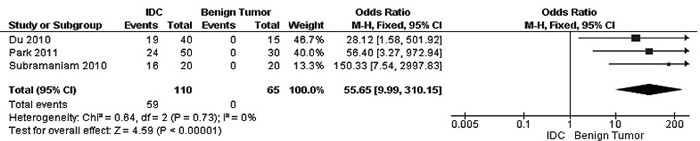
Forest plot for *RUNX3* methylation in IDC and benign tumor The squares represent the weight of individual study in the meta-analysis, the line width indicates the corresponding 95% CI, The diamond represents the pooled OR, and the width of diamond indicates 95% CI. Abbreviations: M-H: Mantel-Haenszel, CI: Confidence Interval, IDC: Invasive Ductal Carcinoma.

**Figure 5:**
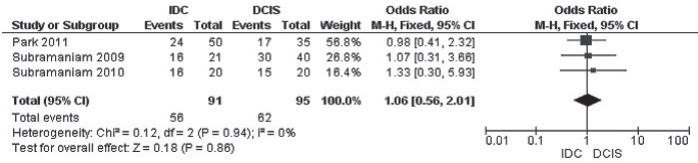
Forest plot for *RUNX3* methylation in IDC and DCIS The squares represent the weight of individual study in the meta-analysis, the line width indicates the corresponding 95% CI, The diamond represents the pooled OR, and the width of diamond indicates 95% CI. Abbreviations: M-H: Mantel-Haenszel, CI: Confidence Interval, DCIS: Ductal Carcinoma *In Situ,* IDC: Invasive Ductal Carcinoma.

**Figure 6:**
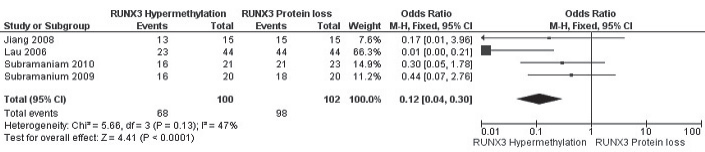
Forest plot for the correlation of *RUNX3* hypermethylation and RUNX3 loss in IDC tumor The squares represent the weight of individual study in the meta-analysis, the line width indicates the corresponding 95% CI, The diamond represents the pooled OR, and the width of diamond indicates 95% CI. Abbreviations: M-H: Mantel-Haenszel, CI: Confidence Interval.

**Figure 7:**
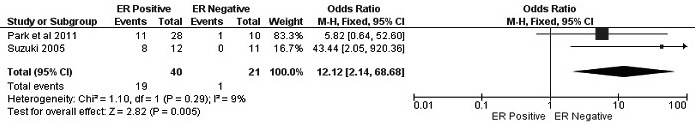
Forest plot for *RUNX3* methylation in ER positive and negative of BC The squares represent the weight of individual study in the meta-analysis, the line width indicates the corresponding 95% CI, The diamond represents the pooled OR, and the width of diamond indicates 95% CI. Abbreviations: M-H: Mantel-Haenszel, CI: Confidence Interval, ER: Estrogen Receptor.

**Figure 8:**
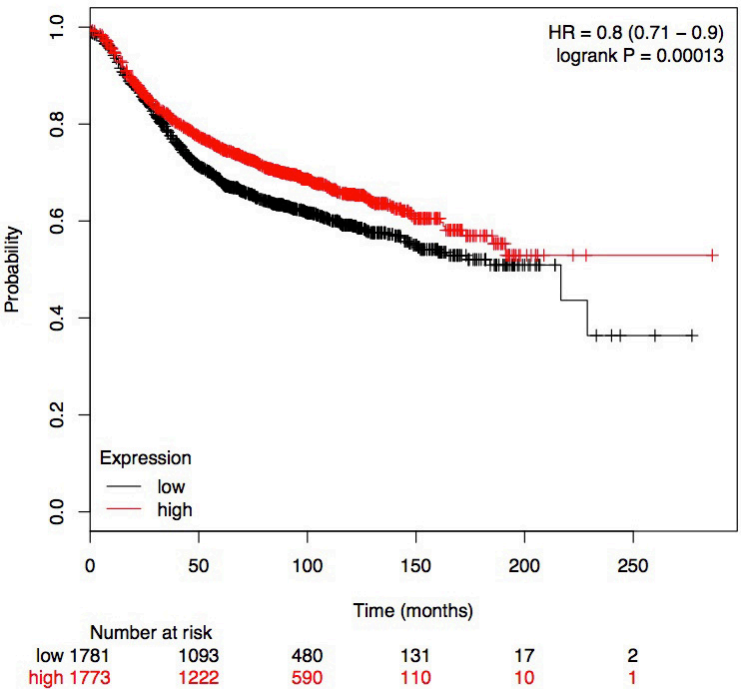
Plot for the relationship of *RUNX3* mRNA expression and RFS in BC patients HR: Hazard Ratio

The frequency of RUNX3 methylation was significantly higher in DCIS than in normal breast tissues and the pooled OR was 50.37 with 95% CI 12.32-205.90, z = 5.46, *p* < 0.00001, I2 = 0%, *p* = 0.76 (Figure 2). RUNX3 promoter in IDC patients was significantly methylated than in normal breast, OR was 22.66 with 95% CI 12.48-41.17, z = 10.25, *p* < 0.00001, I2 = 0%, *p* = 0.43 (Figure 3). In addition, RUNX3 methylation was significantly increased in IDC than in benign tumor, OR was 55.65 with 95% CI 9.99-310.15, z = 4.59, *p* < 0.00001, I2 = 0%, *p* = 0.73 (Figure 4). RUNX3 methylation was not significantly increased in IDC than DCIS, OR was 1.06 with 95% CI 0.56-2.01, z = 0.18, *p* = 0.86, I2 = 0%, *p* = 0.94 (Figure 5). RUNX3 methylation was strongly correlated to RUNX3 loss, OR was 0.12 with 95% CI 0.04-0.30, z = 4.41, *p* < 0.0001, I2 = 47% (Figure 6). In addition, the frequency of RUNX3 methylation was higher in ER positive patients with BC than in ER negative patients with BC (Figure 7). The OR was 12.12 with 95% CI 2.14-68.68, z = 2.82, *p* = 0.005, I2 = 9%, *p* = 0.29. High RUNX3 mRNA expression was strongly correlated to better relapse-free survival (RFS) in all 3554 BC patients (Figure 8).

**Figure 9:**
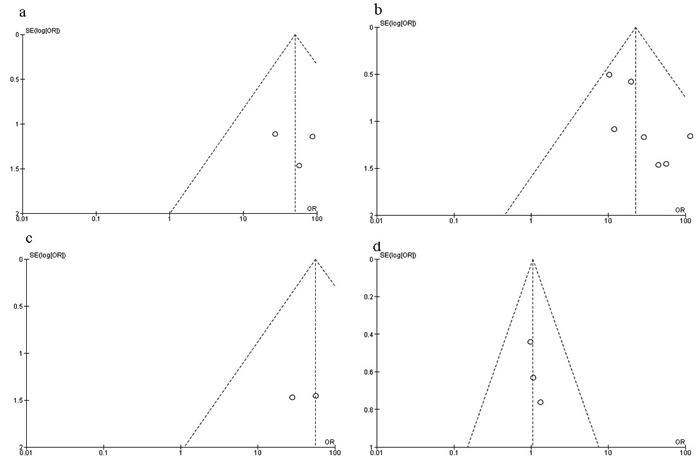
Funnel plot for publication bias **a**. *RUNX3* methylation in DCIS and normal breast tissue; **b**. *RUNX3* methylation in IDC and normal breast tissue; **c**. *RUNX3* methylation in IDC and benign tumor; **d**. *RUNX3* methylation in IDC and DCIS. Y-axis represents the standard error, X-axis represents order ratio, Area of the circle represents the weight of individual study.

The quality of each study was evaluated using the Newcastle Ottawa Quality Assessment Scale (NOQAS). Non-randomized case controlled studies and cohort studies was assigned up to nine points in three domains, 1) selection of study groups, 2) comparability, 3) exposure, and outcomes for study participants. Among studies, five graded 8 points and five graded 7 points. Those studies were of a relatively high quality (Table 2). A sensitivity analysis, in which one study was omitted at a time, was performed to assess the result stability. The pooled ORs were not affected, indicating the stability of present analyses. The symmetry of funnel charts (Figure 9) suggested that there were no publication biases in the meta-analysis of RUNX3 methylation in BC.

**Table 2:** Quality assessment according to the Newcastle–Ottawa scale of the included studies

Author	Selection	Comparability	Exposure	Total score
Park et al[38]	2	2	3	8
Subramaniam et al[39]	2	2	3	8
Subramaniam et al[10]	2	2	3	8
Lau et al[15]	2	2	3	8
Du et al[40]	2	1	3	7
Suzuki et al[11]	2	1	3	7
Li et al[41]	2	2	3	8
Qiao et al[42]	2	1	3	7
Tian et al[43]	2	1	3	7
Jiang et al[16]	2	1	3	7

## DISCUSSION

Several studies have reported the contribution of RUNX3 hypermethylation in BC progression by utilizing small group of patients. To overcome small sizes of individual studies, we conducted the powerful meta-analysis with a total of ten studies and 747 patients, and evaluated the role of RUNX3 hypermethylation in the carcinogenic progression of BC. The pooled OR of RUNX3 methylation in DCIS and NB or benign tumor under the fixed-effects model was 50.37 which indicated the frequency of RUNX3 hypermethylation in DCIS significantly increased compare to NB or benign tumor, the heterogeneity did not show significant difference among studies at Cochran's test, with low I2 index (0%), suggesting RUNX3 methylation is an early event during carcinogenesis which is consistent with the results of the original articles included in present study. Similarly, the hypermethylation rate of RUNX3 in IDC was also significantly higher than in NB or benign tumor. Our results are consistent with previous studies [19]. The RUNX3 gene resides on human chromosome 1p36, a region that genomic deletion frequently happened in various human cancers, including BC [20]. The RUNX3 transcription factor is a downstream effector of TGF-β signaling pathway. TGF-β is activated after binding to Smad 4 (co-Smad) and enter the nucleus. RUNX3 binds R-Smads, co-Smads and p300, a transcriptional co-activator, and fulfills its tumor suppressor activity *via* TGF-β signaling pathway [12]. Xie et al has observed the downstream SMAD pathway stays active in majority of BC cells [21]. The sensitivity to TGF- β was re-established in RUNX3-deficient cancer cells after reintroducing RUNX3 into those cells which showed increased expression of proapoptotic gene BIM [22]. In addition, the status of EGFR, p53 or KRAS in RUNX3 hypermethylation in BC was unavailable, weather RUNX3 silencing contributes to the development of BC concomitantly or independently, further investigations are needed. Previous evidences indicate RUNX family genes regulate cell fate through p53-dependent DNA damage response and/or tumorigenesis [23–24]. Additionally, Omar et al reported that aberrant expression of RUNX3 was not biased toward the EGFR or KRAS mutation pathway in lung adenocarcinoma (ADC), indicating that RUNX3 methylation contributes the development of ADC in an independent of EGFR or KRAS pathway [25].

Recent evidences indicate that RUNX3 hypermethylation attributes to the development of BC through Wnt signaling pathway. Wnt signaling pathway is not only critical for the development of the mammary gland, but also is important for regulating cell proliferation and survival. RUNX3 interacts with β-catenin/TCFs and forms a complex which inhibits the transactivation *via* blocking β-catenin/TCFs DNA binding [26]. Ito et al has observed that RUNX3 down-regulates Wnt signaling by directly inhibiting β-catenin/TCFs in colon cancer and gastric cancer [13]. The activation of the Wnt/β-catenin pathway was observed following knockdown of the tumor suppressor gene phosphatase and tensin homolog (PTEN) in human breast cells [27]. Furthermore, the downregulation of the Wnt inhibitor Secreted Frizzled-Related Protein1 (Sfrp1) was observed in most invasive human BC [28]. Taken together, RUNX3 as a suppressor plays critical role in the development and progression of BC *via* Wnt signaling pathway [29–30]. Therefore, high RUNX3 mRNA expression is associated with better relapse-free survival (RFS) in BC patients (Figure 8).

Kang et al reported 5-aza-2′-deoxycytidine (5-Aza-CdR), a demethylation agent restored the expression of RUNX3, induced apoptosis and inhibited cell proliferation in the breast cancer MCF-7 cell line. [31] In addition, miR-29 family members (which downregulated the DNA methyltransferases DNMT3A and DNMT3B in non-small cell lung cancer [32]) could decrease promoter methylation and increase expression of RUNX3, as a result, those agents potentially suppress tumor proliferation and induced apoptosis. Although more investigation needs to complete, RUNX3 could be a potential therapeutic target for the development of personalized treatment *via* demethylation.

In addition, we found that RUNX3 promoter methylation was not significantly increased in DCIS compared to IDC. This data indicated that RUNX3 hypermethylation may not be required during the progression from DCIS to IDC. Although there was not heterogeneity existed between included studies, further studies with higher power are needed to confirm this point. RUNX3 methylation could also be detected in the sera of patient with BC [33]. More extended studies are needed to investigate the potential value of RUNX3 as a diagnostic marker for BC in future.

We pooled four studies and evaluated the association between RUNX3 methylation and RUNX3 loss, the result showed that OR was 0.12, *p* < 0.0001, I2 = 47%. There was moderate evidence for heterogeneity across studies (I2 = 47%, *p* = 0.13), mostly accounted for by Lau et al which reported lower rate of RUNX3 methylation (52%) compared to other three studies (the rate of RUNX3 methylation arranged from 76% to 86%). Removal this study from meta-analysis reduced the I2 statistic to 0%, OR was changed to 0.32 with 95% CI 0.10-1.03, *p* = 0.06, close to significantly different. Our finding indicated that RUNX3 methylation was correlated with RUNX3 loss, but more studies with a large population need to be completed.

Two studies have shown RUNX3 methylation was higher in ER positive BC patients than in ER negative BC patients. ER signaling plays an important role in the development of mammary gland through the regulation of cell proliferation and apoptosis [34]. Abnormal ER signaling contributes to initiation and progression of BC [35]. Recent evidence showed that RUNX3 inhibits ER signaling through suppressing the transcription activity of ERα and reducing ERα-dependent cancer cell proliferation. Therefore, RUNX3 mRNA high expression was correlated to better overall survival in ER-negative patients, but not in ER-positive patients [19]. Aberrant RUNX3 expression contribute to the development and progression of BC through modulating ER signaling pathway. Overexpression of RUNX3 in BC cells decreases ERα expression, whereas deletion of RUNX3 by siRNA increases ER expression. Expression of RUNX3 is inversely associated with ERα in breast cells lines and human BC tissue [36]. Further investigation with a large population needs to be carried out to confirm this mechanism.

There are some limitations in this meta-analysis. Present results were based on individual unadjusted ORs, while further investigation should be adjusted by other potential risk factors. In addition, most selected studies are from Asia population, thus, the findings of this meta-analysis should be interpreted with caution. All the included studies are observational studies which are well known selection bias and publication bias, as positive results may be more likely to be published than negative results. We only selected relevant studies in English and Chinese, some eligible studies in other languages may be excluded, indicating language bias probably introduced.

In summary, the results of present meta-analysis suggest that the frequency of RUNX3 hypermethylation significantly increased in DCIS and IDC. RUNX3 methylation could be a promising biomarker for early diagnosis of BC. High RUNX3 mRNA expression is correlated to better relapse-free survival (RFS) in all BC patients.

## MATERIALS AND METHODS

### Search strategy and selection criteria

The following electronic databases were reviewed without any language restrictions: PubMed (1966 ∼ 2016), Web of Science (1945 ∼ 2016), EMBASE (1980 ∼ 2016), Cochrane Library Database (1972-2016), CNKI and Google scholar. The following key words were used: “RUNX3 methylation” and “breast cancer”. There were 35 studies identified from PubMed, 56 studies from Web Science, 39 studies from Embase, 432 studies from CNKI. First 300 of 2700 articles were screened from google scholar.

The following were criteria for the inclusion: 1) The studies about RUNX3 methylation and the clinicopathological significance in BC; 2) RUNX3 methylation in prognosis of patients with BC. 21 relevant studies were included for full text review. Among of them, 11 studies were excluded for evaluating RUNX3 protein expression, or using the same population, conference abstracts containing insufficient data, and using cell lines. The variables from 10 related studies were listed in Table 1.

### Data extraction and study assessment

Two authors (DL and YM) performed independent systematic reviews and collected data by using a standardized data extraction form including the following variables: first author's name, year of publication, countries, number of patients, study population, ER status, stage of BC, grade of BC, and RUNX3 methylation rate, RUNX3 expression. Any discrepancies were discussed and reached a consensus for all issues.

### Statistics analysis

Odds ratios (ORs) with 95% confidence intervals were calculated by using a fixed or random effect model. Heterogeneity among studies was evaluated by using the Cochran Chi-square test and qualified by I2 statistics. When the heterogeneity I2 < 50%, a fixed effect model was used, otherwise. When I2 >50%, indicated substantial heterogeneity among studies, a random effect model was adopted. Potential sources of heterogeneity were then investigated using subgroup analysis and meta-regression. The analysis was conducted to compare RUNX3 methylation between DCIS and normal tissue, IDC and normal tissue, IDC and DCIS, RUNX3 methylation in ER positive and native patients. Two-sided statistical tests and *p*-value were used. Relapse-free survival was analyzed by using an online database Kaplan Meier-plotter (cancer survival analysis) (http://kmplot.com/analysis/index.php?p=service&cancer=breast). The database was established by using gene expression and the survival information of 3554 BC patients [37]. Publication bias were evaluated by the funnel graphs. An asymmetric funnel plot in meta-analysis suggests the existence of publication bias. All analysis was conducted with Review Manager 5.2.

## SUPPLEMENTARY MATERIALS FIGURES


